# Perspectives on Genetically Engineered Microorganisms and Their Regulation in the United States

**DOI:** 10.1021/acssynbio.4c00048

**Published:** 2024-04-26

**Authors:** Arik Shams, Alexandria Fischer, Anastasia Bodnar, Melinda Kliegman

**Affiliations:** † Kavli Center for Ethics, Science, and the Public, University of California—Berkeley, Berkeley, California 94720, United States; ‡ United States Department of Agriculture, Washington, D.C. 20250, United States; § Innovative Genomics Institute, University of California—Berkeley, Berkeley, California 94720, United States

**Keywords:** microorganisms, genetic engineering, biotechnology, synthetic biology, regulations, science policy

## Abstract

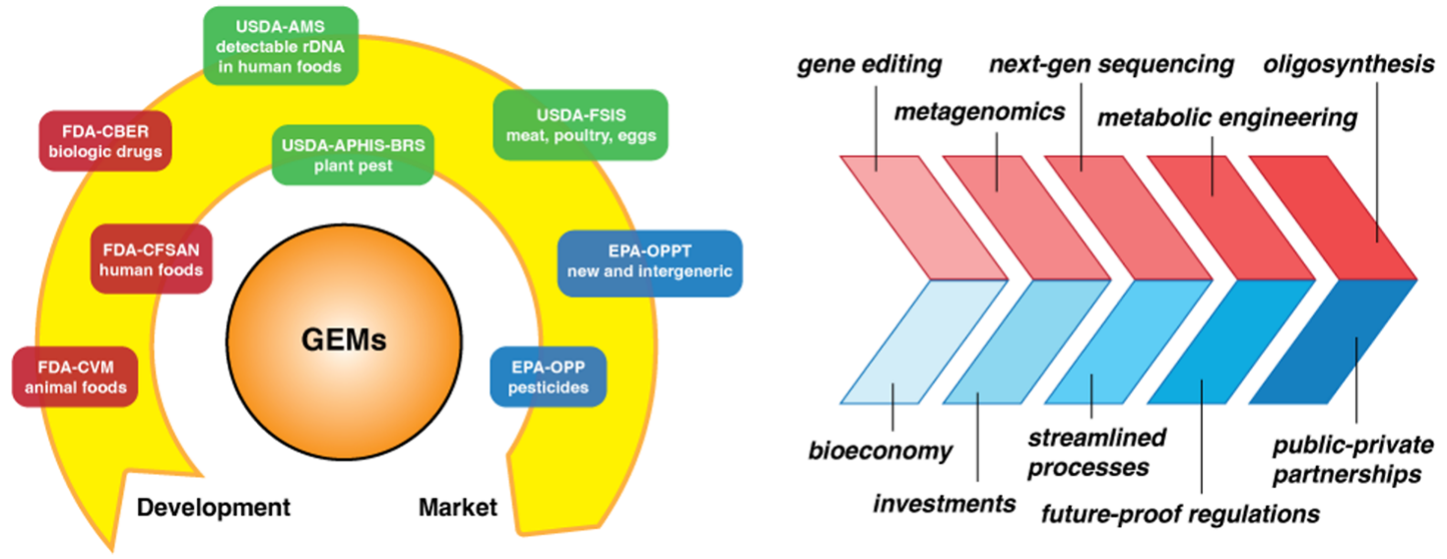

Genetically engineered microorganisms (GEMs) represent a new paradigm in our ability to address the needs of a growing, changing world. GEMs are being used in agriculture, food production and additives, manufacturing, commodity and noncommodity products, environmental remediation, etc., with even more applications in the pipeline. Along with modern advances in genome-manipulating technologies, new manufacturing processes, markets, and attitudes are driving a boom in more products that contain or are derived from GEMs. Consequentially, researchers and developers are poised to interact with biotechnology regulatory policies that have been in effect for decades, but which are out of pace with rapidly changing scientific advances and knowledge. In the United States, biotechnology is regulated by multiple agencies with overlapping responsibilities. This poses a challenge for both developers and regulators to simultaneously allow new innovation and products into the market while also ensuring their safety and efficacy for the public and environment. This article attempts to highlight the various factors that interact between regulatory policy and development of GEMs in the United States, with perspectives from both regulators and developers. We present insights from a 2022 workshop hosted at the University of California, Berkeley that convened regulators from U.S. regulatory agencies and industry developers of various GEMs and GEM-derived products. We highlight several new biotechnologies and applications that are driving innovation in this space, and how regulatory agencies evaluate and assess these products according to current policies. Additionally, we describe recent updates to regulations that incorporate new technology and knowledge and how they can adapt further to effectively continue regulating for the future.

## Introduction

Genetic engineering, the direct manipulation of genomes via recombinant DNA and molecular biology, has catapulted both our understanding and control of complex biological systems into a new “biotechnology era.” Recent advances in genome editing, oligonucleotide synthesis, sequencing, and bioinformatic processing have pushed us into the realm of synthetic biology, or the de novo synthesis of life. These alterations started first in the smallest and most manipulatable forms of life, microorganisms, with their small size and rapid generation times, and has resulted in genetically engineered microbes being used to develop a wide variety of products. Microorganisms are already used in many industries to make high-value chemicals, such as additives, pharmaceuticals, fragrances, and flavors.^1^ Common antibiotics (penicillin, erythromycin, vancomycin, etc.),^2−4^ food additives (vitamins, monosodium glutamate),^5^ and other pharmaceuticals have been successfully produced by microbial biosynthesis at scale. More recently, biomaterials, pesticides, and environmental remediation tools have been developed using engineered microorganisms. Any future that includes genetically engineered microorganisms also necessitates responsible development and regulation. To that end, the regulation of bioengineered products is critical and needs to adapt alongside technological development while being nimble to accommodate products in the pipeline.

### The Future of Microbial Biotechnology: From Research to Regulation

In February 2022, the Innovative Genomics Institute, the United States Department of Agriculture (USDA), and Phytobiomes Alliance jointly hosted a virtual workshop to foster open discussion between regulators, scientists, and developers on the future of genetically engineered microorganisms (GEMs), titled “The Future of Microbial Biotechnology: From Research to Regulation”.^6^ The purpose of the workshop, which was open to the public, was to convene people who research, develop, and regulate GEMs and GEM-based products to develop a better understanding of each group’s approach. Participants highlighted that they use engineering to leverage existing capabilities present in natural microbes, and most participants work with both natural and engineered microbes to achieve similar goals. In some cases, participants remarked that there are few new products coming from the development of wild-type microorganisms and therefore synthetic biology or genetic engineering have good promise.

The workshop came at a critical time of active transformation in the field of biotechnology. Biotechnology, and in particular GEMs, has attracted attention as a solution for intractable problems such as climate change. Climate change necessitates that we quickly employ a broad and diverse range of solutions for emergent global crises: feeding a growing population, loss of biodiversity, greenhouse gas emissions, and fossil fuel dependence.^7−9^ This, coupled with major scientific advances, commercial opportunity, and regulatory updates present a unique moment for developers and regulators alike.

In this review we provide proceedings from the meeting and discuss existing and emerging GEM products. Our scope will be limited to food, agricultural, and environmental applications, as these areas have significantly overlapping statutes and regulatory oversight. We will also discuss U.S. regulatory agencies and policies that may apply to each GEM product, highlight instances where there are regulatory gaps, and make recommendations for cohesive, yet adaptive regulatory coverage of innovative new technologies in the future.

### GEMs: What Are They?

Microorganisms have been controlled and cultivated in the production of food, fuel, and materials for millennia. Even before humans knew of microbes’ existence, they were used to making bread, cheese, wine, and yogurt. In the 1800s, microorganisms’ role in causing disease and food spoilage was discovered, which spurred the race to manipulate them for new applications. Today, genetic engineering of microorganisms to make new products is becoming increasingly common. Throughout this review we use the term “genetically engineered microorganism” or “GEMs” to describe a microscopic organism with a genome directly altered using biotechnological methods.

The term microbe or microorganism itself is not clearly defined scientifically and contains species from all three domains of life (Archaea, Bacteria and Eukarya). It is rather defined *functionally* as “whole organisms which are on the microscopic scale.” Entities around the world define the term differently to suit various criteria, especially with regard to regulatory oversight. The United Nations Food and Agriculture Organization (FAO) for example, defines microorganism as simply “a protozoan, fungus, bacterium, virus, or other microscopic self-replicating biotic entity.”^10^ U.S. regulatory agencies all define microorganisms, with some variation, as yeasts, molds, bacteria, viruses, protozoa, and microscopic parasites, [including] species that are pathogens, that can only be observed through a microscope.^11^

Similarly, the terms genetic engineering and biotechnology are defined differently by different organizations. For example, the US Department of Agriculture (USDA) defines genetic engineering as “manipulation of an organism’s genes by introducing, eliminating, or rearranging specific genes using modern molecular biology methods, particularly those techniques referred to as recombinant DNA techniques.”^12^

The emergence of genome-editing has added to the complexity of these definitions, as it is now possible to make precise deletions or nucleotide changes within a gene. Arguably, these changes are equivalent to those produced though natural processes, or conventional methods such as mutagenesis.^13^ Many governments internationally are adopting regulatory policies that consider such products as conventional; U.S. regulatory agencies are determining how such products fall within existing regulatory processes.

### Regulation of GEMs in the United States

Regulatory oversight of GEMs in the United States involves multiple government agencies, depending on how the microorganisms will be used: specifically, the Animal and Plant Health Inspection Service (APHIS), the Food Safety and Inspection Service (FSIS), and the Agricultural Marketing Service (AMS) in the USDA; the Food and Drug Administration (FDA) in the Department of Health and Human Services (HHS); and the Office of Pesticide Programs (OPP) and the Office of Pollution Prevention and Toxics (OPPT) in the Environmental Protection Agency (EPA). The Coordinated Framework for Regulation of Biotechnology (CF), instituted in 1986, stated that agencies would regulate products of biotechnology using their existing regulatory authorities. Although the CF sought to simplify oversight of biotechnology, these agencies maintain distinct but overlapping roles. As described in the Workshop, the main tenets of the framework are as follows: (1) regulations should focus on the products of biotechnology rather than the process used to make them, (2) regulations should be based on science, and (3) existing statutes are sufficient to review biotechnology products. Since its publication, these tenets of the CF have provided the foundation for U.S. regulation of biotechnology.

The CF has undergone several updates since its initiation, notably in 1992 and again more recently in 2017.^14^ The 2017 update was intended to clarify the roles and specific areas of focus for each agency. In addition to reaffirming the governing tenets described previously, one of the aims of the 2017 CF update was to revamp the framework such that emerging technologies and products could be more easily reviewed and regulated under existing guidelines, in an effort to modernize and “future-proof” U.S. regulation of biotechnology. A unified Web site for the coordinated framework was established after the 2017 update.^15^ Figure 3.1 outlines a simplified process for determining which offices within each agency may regulate a particular GEM.

### US Department of Agriculture

USDA-APHIS is the agency responsible for protecting agriculture and natural resources from plant and animal pests and diseases. Relevant to GEMs, the Biotechnology Regulatory Service (BRS) within APHIS regulates genetically engineered organisms that are or may be plant pests. Plant pests are defined by APHIS under 7 CFR 330.100 as “Any living stage of any of the following that can directly or indirectly injure, cause damage to, or cause disease in any plant or plant product: A protozoan, nonhuman animal, parasitic plant, bacterium, fungus, virus or viroid, infectious agent or other pathogen, or any article similar to or allied with any of the foregoing.”^16^ A GEM may be regulated by BRS if it is classified taxonomically as a plant pest, contains DNA from a plant pest, or will be used to biologically control plant pests. In these cases, BRS regulates the interstate movement and importation, as well as potential environmental release of the GEM, and any developer that is planning to do so must obtain a permit from BRS.

FSIS within USDA is responsible for ensuring that meat, poultry, and egg products are safe, wholesome, and properly labeled. FSIS reviews new technologies used in food production, irrespective of whether a GEM is used. FSIS oversees GEMs used in processing, packaging, or other uses associated with meat, poultry, and egg products.

The USDA Agricultural Marketing Service (AMS) implements the National Bioengineered Food Disclosure Standard, which requires food manufacturers, importers, and certain retailers to disclose information about whether food offered for retail sale is bioengineered (BE) or uses BE food ingredients. The Standard is designed to provide consumers more information about their food. The Standard defines bioengineered foods as those that contain detectable genetic material that has been modified through in vitro recombinant DNA (rDNA) techniques and for which the modification could not otherwise be obtained through conventional breeding or found in nature.^17^

### Food and Drug Administration

As the primary regulatory agency for food safety in the U.S., FDA oversees any GEM product or additive that is be intended for human consumption. There are multiple avenues employed by FDA for regulation of GEMs, depending on the product, and as such FDA uses multiple terms and definitions for these products.

The FDA Center for Food Safety and Applied Nutrition (CFSAN) ensures safety of foods and food additives and undertakes a review process to determine whether products are safe or “Generally Recognized As Safe” (GRAS). For a product to be GRAS, it must satisfy any of the following criteria: scientific data about the substance must be widely available and there must be consensus among qualified experts that the substance is safe for consumption, or the substance must have been commonly in use since 1958 and therefore its safety determined through common use.^18^

Any GEM or GEM-derived food additive must be determined safe either before it goes to market, and the data provided to CFSAN for approval. If a GEM is present in food, CFSAN reviews the food for safety, the same as they would for any other food.^19^ In the case of enzymes derived from GEMs, CFSAN recommends (but does not require) that the developer provide the detailed identity of the microorganism used to make the enzyme, including genetic modifications to the gene expressed and the host organism, as well the processes to obtain the microorganism and the enzyme. FDA will then make a determination on whether the additive is approved for market.^20^

Foods may additionally require proper bioengineered labeling as described above—an example of how more than one agency can be involved in the regulation of one product.^19^

In addition, the FDA’s Center for Veterinary Medicine (CVM) regulates GEMs that are used in animal feed, such as feed additives intended to reduce enteric methane emissions in cattle. This may include animal feed that is itself genetically altered (GEM additives) or derived from products that have been engineered (plants treated with a GEM pesticide). The FDA’s Center for Biologics Evaluation and Research (CBER) also regulates GEMs as they relate to human medicine, such as vectors for gene therapy, and has published industry guidelines for their development;^21^ however that is beyond the focus of this article.

### Environmental Protection Agency

The EPA also has multiple offices and statutes relating to the regulation of GEMs in scenarios where environmental release may be a concern. The EPA Office of Pesticide Programs (OPP) contains several functional offices that regulate biopesticides including GEMs and GEM-derived substances. The EPA’s authority also extends to food and sets limits on amounts of pesticide residue allowed to remain on food before sold to consumers. Under the Toxic Substances Control Act (TSCA), the Office of Pollution Prevention and Toxics (OPPT) regulates several classes of substances, which includes bioremediation agents, biofertilizers, and compounds used in the production of biofuels—all of which may contain GEMs or GEM-derived substances. Thus, the EPA has broad authority over any GEMs that may be intended for (or run the risk of) environmental release.^22^

The Biopesticides and Pollution Prevention Division (BPPD) within OPP oversees biologically based pesticides, which includes chemicals derived from GEMs or GEMs themselves (e.g., bacteriophages and fungicides). Like any new pesticide, a biopesticide containing or made from GEMs must provide biological safety data for both human and environmental exposures, as well as potential societal and economic impacts to BPPD for approval. Additionally, OPP enforces a maximum limit for any biopesticide residue remaining on food, and places special consideration on infectivity, toxicity, and pathogenicity of a GEM-based pesticide.^22^

Under TSCA, virtually any GEM will be regulated as a new “substance” as its genetically recombinant composition excludes it from TSCA’s Inventory of Chemical Substances. As a result, EPA-OPPT regulates any new microorganisms developed via genetic engineering, even if the product is also regulated by USDA, unless otherwise exempt.

In 1997, the EPA published detailed guidance and rules for development and use of Microbial Products of Biotechnology under TSCA. While minor updates and exemptions have been made since then,^23,24^ this rule remains largely unchanged. Any developer making a GEM product for environmental release must register with the EPA, file a Microbial Commercial Activity Notice (MCAN), and follow EPA’s evaluation standards throughout testing and development.

## GEMs in Food and Agriculture

GEMs used in food and agriculture include food ingredients and processing aids, feed additives, microbes to alter plant–microbe interactions or to improve nutrient availability, and biological pesticides. Although there are many applications being developed in the early discovery phases, below we focus on GEM products that are either in or nearing the commercialization stage.

### Food Ingredients and Processing Aids

One of the areas where there has been significant use of microbial biotechnology is in food enzymes and additives. Biotechnology has long been used to make chymosin, a key enzyme in rennet, which is used to make most hard cheeses on the market. Another dairy product, yogurt, was the source of the discovery of CRISPR systems, as scientists sought to engineer bacteria at Danisco.^25^

There has been a recent boom in the use of genetic engineering to make beer, with some companies such as Omega Yeast^26^ and Berkeley Yeast^27^ providing new strains. To date, there have been several GRAS notices submitted to the FDA by developers of engineered brewer’s yeast strains, and most of them have elicited a “no questions” response from the FDA.

Another new area is that of replacement meat using microbial derived products as a means of improving sustainability related to the production of GHGs during meat production. During the Workshop, the Good Food Institute, an advocacy organization focused on alternative proteins, stated that three-fourths of the land used for agriculture is dedicated to growing feed crops or grazing ruminants.^28^ Precision fermentation with genetically engineered microbes can produce ingredients for meat alternatives. Some companies developing these products include Motif (bovine heme, proteins found in eggs and milk), Perfect Day and Nobell (dairy alternatives), EVERY Company (pepsin and egg alternatives), and Impossible Foods (legume hemoglobin).

As described in the previous section, food ingredients that are made from or contain GEMs would likely be regulated by HHS-FDA-CFSAN and would require a GRAS notification or a food additive petition. If the GEM is in the final food product, then that food product may also be required to display a bioengineered food disclosure under the National Bioengineered Food Disclosure Standard.^17^

### Nutrient Availability

Microbes can be found living in close association with plants and are required for many key plant metabolic functions.^29−31^ Nitrogen (N), phosphorus (P), and potassium (K) are all essential macronutrients for plant growth and function that are typically applied to agricultural fields as synthetically derived fertilizers. In all three cases bioavailability to the plant is mediated by microorganisms. Replacing or reducing the need for synthetic fertilizer has been one area of intense focus for genetically engineering microorganisms.

Nitrogen fixation in legume plants occurs through a symbiotic relationship with micro-organisms that fix atmospheric nitrogen and provide nitrogen in the form of ammonia to plants. While there have been improvements to this process in legumes^29,32^ which already form symbiosis with nitrogen fixing bacteria, engineering approaches have had limited tractability in cereal crops which provide a majority of the world’s calories and where most of the world’s synthetic nitrogen fertilizer is directed.

To date, limitations in nitrogen availability have been circumvented through the industrialized Haber-Bosch process which uses high temperatures and pressures to combine atmospheric nitrogen with hydrogen gas to form ammonia.^33^ In addition to the use of energy and release of greenhouse gases (GHG) during the manufacturing process, globally ∼50% of N applied to agricultural fields is lost to the environment;^34^ industrially synthesized N is more prone to leaching and volatilization as nitrous oxide (N_2_O) after application than biologically fixed nitrogen. Thus, improving N fixation by microorganisms presents a unique opportunity to improve environmental sustainability and GHG emissions.

During our workshop we heard from both Joyn Bio and Andes Ag, two biotechnology companies working to replace synthetic nitrogen fertilizers using GEMs. Both companies aim to alter the genes of naturally occurring microbes involved in nitrogen fixation with a focus on engineering symbiosis with cereal crops. In the case of Andes Ag, microbes are delivered to farmers on seeds to improve efficacy by ensuring interaction with the plant and reduced competition with soil microbes already present.

One product already on the market is PROVEN by Pivot Bio which reduces the use of nitrogen fertilizers by bioengineering microbes. Pivot Bio screened naturally occurring bacteria for nitrogen fixation qualities then used gene editing to continuously activate genes involved in nitrogen production. These bacteria that associate with plant roots are applied with corn seeds upon planting.^31,35^ While Pivot Bio uses genetic engineering, all the genes involved are endogenous to the species of bacteria and as such are not considered transgenic. Other organizations and initiatives that aim to engineer microbes to reduce the need for nitrogen fertilizers include the Engineering Nitrogen Symbiosis for Africa initiative.

In addition to nitrogen, both phosphorus and potassium are key to plant metabolism and physiology. Both are present in soil but only small amounts are in forms that are bioavailable to plants; they can be made more bioavailable via microbial processes.^36,37^ Although there are references in the scientific literature to engineering microorganisms to provide more P and K, we are not aware of commercial attempts being made at this time. We anticipate efforts around increasing P and K bioavailability in the future.

These GEM based fertilizers would likely be regulated by the EPA-OPPT because they would be considered new chemicals or pathogens that may be released into the environment. Additionally, if the fertilizers met the definition of the plant pest, these products would also need a permit for environmental release from USDA-APHIS.^22,23,38^

### Pesticides

Biological pesticides are an important part of modern agricultural practices. Prevalent in the organic sector, biopesticides are attractive due to their specificity, low toxicity to nontarget species, and high biodegradability when compared to chemically synthesized broad spectrum pesticides.^39,40^ The strain *Bacillus thuringiensis* (Bt) in particular is one of the most widely used biopesticides and has been applied as a commercial product against lepidopteran pests, as it produces proteins that impair their digestive function.^41^ A common approach has been to integrate specific genes from *B. thuringiensis* into the DNA of crop plants to reduce the need for externally applied insecticides.

One example of a microbial biopesticide is a fungicidal amoeba developed by French company Amoéba. This product has been approved for field trials in Europe. This engineered amoeba, *Willaertia magna* C2c Maky, feeds on the fungi that cause wheat rust, and has similar efficacy to chemical fungicides, according to the company’s press release.^42^ Another commercial product, Velifer by BASF, is a strain of the fungus *Beauveria bassiana* that is used as a biopesticide against many insects and phytopathogenic bacteria. Other such fungi are being researched as biocontrol agents that would be less toxic to nontarget organisms than conventional chemical pesticides.^43^

The examples of the GEM based pesticides listed above would likely be regulated by EPA-OPPT because they are biologically based pesticides that are derived from a GEM. They may also be regulated by USDA-APHIS if they meet the definition of a plant pest. In the case of these examples, the GEMs do not remain in the final food product; however, if a similar product was developed that did remain in the final food product, then the GEM may be additionally regulated by HHS-FDA.

## Biomanufacturing and the Environment

Synthetic biology and engineering of microorganisms can offer many benefits in the fields of biomanufacturing and industry that cannot be achieved by conventional petrochemical or organic synthesis-based methods.

### Fuels and Commodity Chemicals

Mass production of chemically simpler chemicals like ethanol and nitrogen fertilizer has traditionally relied on organic synthesis, often from petrochemicals as input. For many commodity chemicals, it has been difficult for microbial fermentation to reach carbon parity with organic synthesis as the products from heterotrophs like yeast are often reduced, lower-carbon products (e.g., ethanol) derived from higher-carbon raw materials (e.g., starch).^44^ Additionally, the production of CO_2_ as a byproduct of fermentation makes it difficult to lower the carbon footprint. However, these issues are solvable in large part by genetic engineering of industrial microbes. Researchers have exploited the natural metabolic pathways of autotrophs like *Clostridium autoethanogenum*, which uses CO_2_ to make acetate, a precursor to acetone and isopropanol.^45^

As the biotechnology and green industries boom, scaling up microbial production of commodity chemicals are becoming more and more economical and carbon-neutral. Existing industrial biotechnologies, even today, promise to make significant reductions in GHG emissions, nonrenewable energy consumption, and losses in efficiency.^46,47^ Many companies have developed new genetically engineered microbes to improve fermentation efficiency, use novel raw materials, reduce carbon footprints, and redirect biological byproducts to more downstream biosynthetic production streams.

LanzaTech, for example, uses proprietary GEMs that convert concentrated waste gases at industrial facilities to make several products, including plastics and biofuels. By redirecting efflux from steel mills, LanzaTech can use concentrated carbon dioxide and hydrogen gases as input to improve efficiency and scale. Acetone and isopropanol traditionally rely on cracking or reforming propene, which is very energy intensive. Even conventional fermentation using sugar feedstocks is inefficient, as sugars are relatively complex molecules which release CO_2_ as a byproduct of lysis. Gas fermentation is a more efficient alternative since it recaptures waste CO_2_ and other hydrocarbons to use as feedstock. Reengineered *C. autoethanogenum*, which is already capable of producing ethanol via fermentation, can redirect ethanol production to that of acetone and isopropanol.^48^

### Materials

Microorganisms are increasingly being used to make a variety of materials, such as plastics, fabric, building materials, coatings, etc. Plastics are one of the more sought-after materials for bioproduction, owing to the enormous ecological costs associated with conventional petroleum-based, nonbiodegradable plastics that are ubiquitous in human society. Bioplastics have several advantages over conventional plastics: their production is not dependent on petroleum, they can be degraded by the environment over time, they can be biocompatible (nontoxic), and they can often use existing organic or industrial wastes as feedstocks.^49^ One of the most well-known forms of bioplastics is polyhydroxyalkanoate (PHA), which is made by more than 300 species of prokaryotes^50^ in the form of granules within their cells. The range of sizes (chain length) of PHAs and the granules’ macromolecular structures allow a diverse set of PHA-derived oligomers to be created. PHAs have similar chemical properties to conventional plastics and are easily biodegraded into simpler carbon compounds by certain bacteria. However, there are still significant costs associated with making PHAs in bulk, due to their extraction and purification costs.

More recently, polylactates have emerged as another promising Bioplastic, similar in properties to PHAs, but with a simpler synthetic pathway. Polylactates are derived from—and broken down into—lactic acid, which is a common metabolite in most chemoautotrophs. Several engineered microbes have been developed that can make PLAs, including *E. coli*(51) and cyanobacteria.^52^ Additionally, these GEMs can be grown in large quantities, and use plant biomass and industrial waste gases as feedstock, which can help offset the costs associated with making bioplastics. One company, Neste, uses biomass from waste feedstocks and oil byproducts to make a variety of bioplastics.^53^

Another biomaterial that has been well-studied and is now being tested in the field is biocement, which is cement derived from microorganisms by precipitation of calcium compounds.^54,55^ These materials are aimed at replacing conventional Portland cement manufacturing and usage. The manufacturing process for Portland cement is carbon-heavy, producing 8% of global CO_2_ emissions to power kilns used to bake the cement precursors.^56^ By contrast biocement can potentially be carbon-negative since it sequesters CO_2_ from the air and groundwater to make cementitious materials. The process of biocementation can also be done in situ, which involves inoculation and culturing microbial colonies on soil that needs to be strengthened. In addition to being released into the environment, biocement can itself be composed of dried microbial biomass, so there are regulatory concerns by the EPA. A bioconstruction company called bioMason uses soil microorganisms to produce calcium carbonate as a cementitious material which can then be used to build biologically inert products like tiles, walls, foundations, etc. In our workshop, bioMason stated that government regulations are a key factor in deployment of their product, especially as they develop higher-performing GEMs in the future.

Fuel or materials manufactured by engineered microorganisms are not themselves regulated as “genetically engineered”. As described above, GEMs would only be regulated by USDA-APHIS if they meet the definition of a plant pest. And, since they are not food products, they are exempt from HHS-FDA-CFSAN oversight. The proprietary GEMs, however, are subject to regulations under the EPA-OPPT. They must undergo a risk assessment by the OPPT to ensure that the GEMs are nontoxic, are not pathogenic to plants and animals, and are safeguarded against accidental release. For environmental safety, the EPA evaluates many GEM products designed to be released to the outside world.^24^

### Bioremediation

GEMs also have a role to play in bioremediation from degrading hydrocarbons, pesticides, plastics, and heavy metals. Besides degrading the usual organic matter and returning nutrients to the soil, GEMs can break down xenobiotic compounds as well.^57^ Several microorganisms have shown broad efficacy against toxic pesticides like profenofos, pyrethroids, and endosulfan.^58−60^ They have also been successful in treating soils contaminated with toxic hydrocarbons^61,62^ and heavy metals.^63^ In the case of plastics, diverse microbial communities have been shown to be most effective for degrading the wide range of polymers present in environmental plastic waste.^64,65^

While the success of wild-type microbial isolates is promising, GEMs open new possibilities for bioremediation. The range of chemical contaminants that are biodegradable could be vastly improved if existing microorganisms could be purpose-built for compounds like toluene, xylene, and salicylate, that would normally be toxic to endogenous microorganisms.^7,66^ Modern technologies like directed evolution can accelerate the search for engineered bacteria that are capable of new, niche chemistries for specific compounds, as was performed for the biodegradation of the pesticide atrazine.^67^ More recently, increasing levels of per- and polyfluoroalkyl substances (PFAS) in the environment have raised ecological and human health concerns, and several detection and remediation approaches using biological pathways have been studied.^68,69^ While complete biodegradation of PFAS remains elusive, the enzymatic pathways investigated so far show great potential for optimization, and offer scalable strategies to remove PFAS from the environment.^68−71^

As is the case with bioconstruction, bioremediation also involves environmental release of GEMs, and as such may be regulated by EPA-OPPT and USDA-APHIS.

## Government Actions to Date

In 2012, the White House released the National Bioeconomy Blueprint, which laid out several long-term goals for U.S. investment into biotechnology for the 21^st^ century.^72^ At that time, the U.S. government identified several trends regarding biotechnology in the scientific, commercial, and public opinion spheres. In the areas of energy, agriculture, and environment, climate change was identified as a potential crisis; each relies on limited resources to serve a growing national and global population.

In 2015, the White House issued a memorandum directing regulatory agencies to update the Coordinated Framework to clarify current roles and responsibilities, and to prepare for future products of biotechnology.^73^ This resulted in a National Strategy for Modernizing the Regulatory System for Biotechnology in 2016 and an updated Coordinated Framework in 2017.^14,74^ However, little has changed with respect to how the agencies coordinate to address their overlapping authority.

In 2022, the White House announced the National Biotechnology and Biomanufacturing Initiative (NBBI), as part of an executive order to adopt a “whole-of-government approach to advance biotechnology and biomanufacturing towards innovative solutions in health, climate change, energy, food security, agriculture, supply chain resilience, and national and economic security”.^75^ This initiative promises investments into research and development, market expansion, training, data sharing, and modernization of the U.S. bioindustry. Overall, this initiative represents a significant commitment by the U.S. government to bring its agencies and offices up-to-speed and in step with the private biotechnology sector, which has been moving at a rapid pace with emerging technologies. Many of the issues the NBBI targets are familiar, having been specified in the 2012 Blueprint, but have yet to be realized.

Importantly, and relevant to this review, the 2022 executive order includes modernizing and streamlining the U.S. regulatory framework for biotechnology products—a longstanding goal for the government. As a result, the regulatory agencies conducted stakeholder outreach and published a report on their findings related to ambiguities, gaps, and uncertainties in biotechnology regulation.^76^ They also published plain language information about the biotechnology regulatory system.^77^

## Future-Proofing Regulations

**Figure 1:**
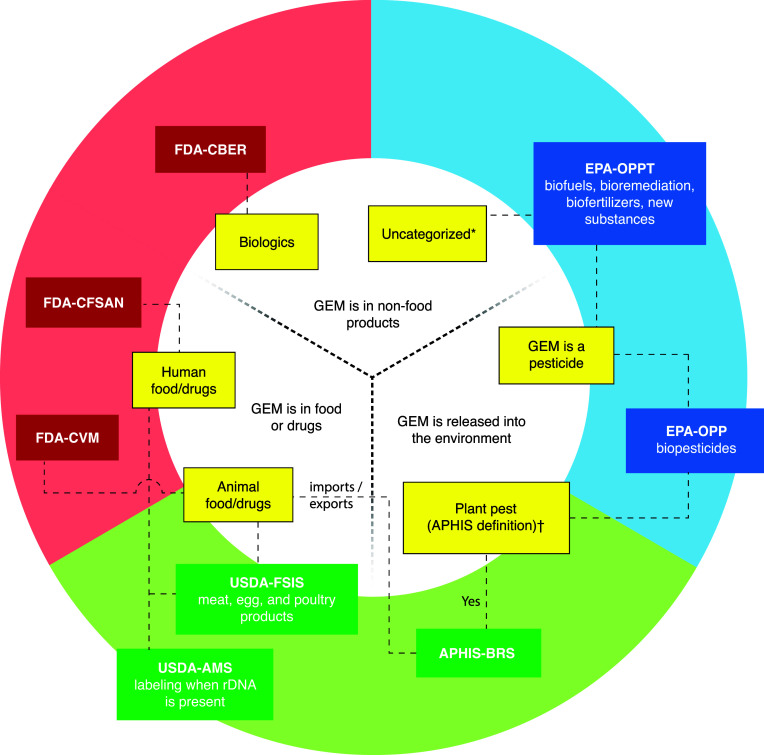
Chart outlining U.S. regulatory agencies that may regulate specific GEMs. A given GEM may be regulated by one or more agencies. Abbreviations for offices are as follows.Department of Health and Human Services (HHS)Food and Drug Administration (FDA)Center for Food Safety and Applied Nutrition (CFSAN)Center for Veterinary Medicine (CVM)Center for Biologics Evaluation and Research (CBER)US Department of Agriculture (USDA)Animal and Plant Health Inspection Service (APHIS)Biotechnology Regulatory Service (BRS)Food Safety and Inspection Service (FSIS)Agricultural Marketing Service (AMS)Environmental Protection Agency (EPA)Office of Pesticide Programs (OPP)Biopesticides and Pollution Prevention Division (BPPD)Office of Pollution Prevention and Toxics (OPPT)^*^This group regulates intergeneric GEMs that do not fit into other regulatory categories and are therefore subject to environmental release safety standards as determined by EPA.

What is evident from the Future of GEMs Workshop is that the complex regulatory landscape can be daunting. Developers of GEMs may need to consult with up to eight agencies and offices (Figure 1), comply with up to 15 different laws, and follow many different regulations and guidelines that may apply to their product (Table 1). This complexity also slows response when new technologies arrive—one agency may quickly produce guidance documents in relation to a new technology while others may take additional time. One approach to streamlining this system could be the creation of a cross-organization panel or review body specifically designed to periodically review new innovations in the pipeline and coordinate agency responses.

**Table 1:** Non-comprehensive List of Laws or Statutes and Their Administering Agencies That May Affect Regulatory Policy for the Development, Distribution, And Sale of GEMs in the United States

law or statute	administering agency(ies)	description
Code of Federal Regulations 7 CFR Part 340	USDA	Regulates movement of any genetically engineered organism, including plants, animals, and microorganism. Defines various biotechnology terms. Grants USDA-APHIS broad authority to evaluate safety and efficacy of engineered products. Establishes SECURE Rule (updated to Biotechnology Regulations).
Plant Protection Act (PPA)	USDA	Regulates the introduction, importation, interstate movement, and release into the environment of genetically engineered organisms that may pose plant pest risks.
Animal Health Protection Act (AHPA)	USDA	Regulates animals or insects that pose a risk to animal health. Restricts imports or entry of any animal, article, or vector if it is deemed to pose a risk for spreading a pest or animal disease. Includes genetically engineered insects, animals, and microbes.
Agriculture Risk Protection Act of 2000	USDA	Revises federal crop insurance program and provides emergency agricultural assistance in the case of natural disasters. Authorizes USDA to restrict plant pests, animals, biological control agents, and invasive species if they pose a threat to the agriculture industry.
Virus-Serum-Toxin Act	USDA	Restricts the preparation and sale of any harmful agents such as viruses, toxins, and other similar products.
Federal Food, Drug, and Cosmetic Act (FFDCA)	FDA	Passed in 1938 to replace the 1906 Pure Food and Drug Act. Broadly authorizes FDA to oversee the safety and labeling of foods, including those derived from biotechnology.
Public Health Service Act (PHS Act)	US Public Health Service (contains multiple HHS agencies including FDA)	Authorizes agencies to prevent the introduction, transmission, and spread of communicable diseases from foreign countries.
FFDCA Section 408	EPA	Extends the FFDCA; sets maximum limits for pesticide residues on foods to be enforced by EPA.
Federal Insecticide Fungicide and Rodenticide Act (FIFRA)	EPA	Regulates all pesticides, insecticides, fungicides, etc., and requires them to be registered and evaluated for safety. Includes engineered pesticidal organisms or their derivatives.
Toxic Substances Control Act (TSCA)	EPA	Regulates several classes of substances, which includes bioremediation agents, biofertilizers, and compounds used in the production of biofuels. Under TSCA, EPA acts as the main regulating body for any previously unknown or uncategorized substance introduced to the environment.
Clean Air Act (CAA)	EPA	Authorizes EPA to protect and improve air quality, reduce pollution, and regulates emissions from industry, agriculture, and vehicles.
Clean Water Act (CWA)	EPA	Regulates water quality and pollution; sets quality standards for water and regulates wastewater discharge.
National Environmental Policy Act (NEPA)	Multiple	Requires federal agencies to evaluate and report on environmental impact for any major policy change or adoption.
Endangered Species Act (ESA)	US Fish and Wildlife Service (FWS), US National Oceanic and Atmospheric Administration (NOAA) Fisheries Service	Requires federal agencies to ensure the protection and conservation of endangered species and their habitats in any action they authorize, fund, or perform.

In other countries, having just one biotechnology agency has also proven to be a workable approach (e.g., Kenya, and Argentina).^78,79^ While the myriad of laws in the U.S. are likely to remain in the future, having a centralized agency to interpret those laws and/or only one application to submit for developers could go a long way in streamlining the process, leading to faster decisions on how new technologies will be treated in the future.

In addition to the complexities of the approvals process, another major issue is obsolescence of current regulation. Despite incremental updates, the Coordinated Framework is nearly 40 years old, and not made for the pace of the current bioindustry. Many new products do not fall into the application spaces which the agencies have historically reviewed; as an example, some new applications for GEMs may focus solely on sustainability end points with the goal of being deployed in natural environments for climate applications. As molecular biotechnology continues to advance, our ability to make genetic alterations become both more precise and ambitious. Current definitions of genetically modified organisms are not on the same continuum: they are inherently categorical and do not accurately represent the nature of the modifications made, and how the end-product is “different” from the starting wild-type organism.

In 2020, USDA-APHIS released an overhaul of their Biotechnology Regulations (previously known as the SECURE Rule), to exempt certain plants that were produced by gene-editing. Edited plants whose genomes have been altered by native cellular repair mechanisms, or contain only a single base-pair substitution, or has received a gene that is already a known allele in the plant’s gene pool are exempt from APHIS regulations.^80^ We recognize this change as an important and forward-thinking change based on current scientific understanding, which will likely have a positive impact on development of new crops and plant products. We also note that the updated rule still leaves open the possibility of new edited products avoiding regulatory triggers while still being phenotypically the same as products made with older, conventional engineering approaches. In fact, APHIS recently proposed five additional categories of genetic modifications that would be exempt from regulation by APHIS.^81^

It is important to note that regulations are often slow to adapt by design, to avoid different standards for different products. Notably, the SECURE Rule has had many proposals to change the regulatory exemptions over decades that failed before the update for edited plants. However, the landscape has changed such that innovation is no longer merely a business edge: climate change, overpopulation, food scarcity, ecosystem collapse, pandemic threats, and more necessitate innovation for survival. Regulations need to be more dynamic, adaptable, and leaner, namely less bureaucratic.

Conversely, product development should also happen with an eye toward regulations. Regulations can serve a role in focusing effort and resources to create commercially viable products. Additionally, they can create an incentive structure to steer development toward addressing societal problems such as climate change, sustainability, and public health. The European Food Safety Authority, for example, recently released a draft proposal to deregulate certain plant products created via gene-editing that improve crop resilience, nutrient use, and yield, in an effort to accelerate research in those areas of critical need.^82^ The proposal explicitly cuts down on red tape associated with evaluating genetically engineered plants, thereby potentially clearing the way for many beneficial products already in development. The proposal also eases regulations on plants that have very small genomic edits and are thus difficult to differentiate compared to conventionally bred plants. These new rules will have a drastic positive impact on the research and innovation landscape of European Union member countries and developers.

## Conclusions

Since the establishment of the Coordinated Framework in 1986, biotechnology regulation has been somewhat at odds with scientific progress. This disharmony is unavoidable: scientific and technical breakthroughs happen continuously, exponentially, and often unpredictably, while statutes and regulations change incrementally over years. Ensuring the safety of any biotechnology product being made available to the public requires careful evaluation, and as a result regulations almost always have to catch up to the pace of technology. In recent years biotechnology has experienced rapid advancements, thanks to advances in gene-editing, genetic sequencing, nucleic acid synthesis, data science, and artificial intelligence, the combinations of which have resulted in an unprecedented capacity for developers to bring new products to the market. Regulatory oversight of these products, however, is still slow and laborious, and in need of harmonization.

In our workshop, one of the major takeaways was the critical need for more clarity and synergy across all the regulatory protocols contained within the Coordinated Framework. Simultaneously, there is a need for evaluation processes to be more adaptable and nimbler so that approvals can be granted or denied quickly. In many cases, the long and multistep approvals process spanning different agencies and offices are a major hindrance for startups and academic developers to get a product to market.^83^ In the current environment, many promising advances in GEM products are coming from biotechnology startups with short funding runways, for whom laborious approvals processes are not viable.

At the same time, agencies must be prepared to evaluate new products efficiently and effectively for safety, not just for human health but environmental impact as well. As the world grapples with macro-level crises like climate change and food insecurity, and as our ability to manipulate microorganisms and microbial communities become more sophisticated, the lines between public and environmental health blur. Better collaboration between regulatory agencies is required for a more holistic oversight process. One of the key ways in which this could be implemented is a unified submissions process for market approval. Additionally, improving transparency in approval timelines will greatly benefit the developers, public, and other stakeholders, as well as ensure accountability for agencies and developers alike.

Deliberate, coordinated action is key to mitigating many of the problems in our uncertain future. In matters like global food systems and climate adaptation, no one technology will be the solution, but biotechnology will continue to play a major role. Large-scale government support and buy-in from all sectors of industry are necessary to find and implement effective regulatory solutions.
