# Correction to “Perspectives on Genetically Engineered Microorganisms and Their Regulation in the United States”

**DOI:** 10.1021/acssynbio.4c00325

**Published:** 2024-05-18

**Authors:** Arik Shams, Alexandria Fischer, Anastasia Bodnar, Melinda Kliegman

The original article cites the product *Willaertia magna* C2c Maky by Amoéba as an engineered fungicide (ref 42).^1^*Willaertia magna* C2c Maky is not an engineered microorganism; the product is derived from the cultivated natural strain of the organism and thus should not be considered a GEM.^2^ The product has been approved for use in the United States by the EPA to control a number of fungal diseases.^3^ We apologize for the error.
